# Supplementary material for: A presaccadic perceptual impairment at the postsaccadic location of the blindspot
Source: PLoS One. 2023 Sep 14;18(9):e0291582. doi: 10.1371/journal.pone.0291582 (PMC10501568; doi:10.1371/journal.pone.0291582)
Supplement: Attachment — Submitted filename: Response to Reviewers v2.docx [file pone.0291582.s001.docx]

Response to Reviewers

*** Line 161 : « before the saccade goal was masked », the saccade goal is never masked, do you mean the discrimination target instead ?**

Thank you for spotting this, we did indeed mean the discrimination target. This has been corrected.

***It is written in the abstract that « There was a significant perceptual impairment at the post-saccadic location of the blindspot relative to the contralateral post-saccadic location of the blindspot and the post-saccadic location of the blindspot in a no-saccade control condition ». It seems then contradictory to read line 169 : « Holm-Bonferroni corrected paired samples t-tests showed that performance at the Blindspot FF was significantly worse in the Saccade condition than in the Fixate condition (64.% vs 82%, t(7) = 4.33, p < .01, d = 1.53). No other comparison reached statistical significance ». May be the stats mentioned in the abstract correspond to ANOVAs and post-hoc tests made with separated temporal bins ? Please provide these post-hoc tests…**

Thanks for pointing this out. When reporting the results on (line 169), we intended to refer to the outcome of 4 paired-samples t-tests comparing performance at each of the 4 locations in the saccade and fixate conditions, not the full suite of post-hoc tests. We agree this is potentially misleading as the reader might reasonably assume that ‘no other tests…’ implies that we ran all possible combinations of posthoc tests. We had also intended to report the contrast between the blindspot FF and the Contralateral to Blindspot Future Field separately, but accidentally omitted it. We have therefore edited the text (lines 164-177) as to be more precise in the reporting of the results, and include the analysis comparing Blindspot FF with Contralateral to Blindspot Future Field referred to in the abstract. We have also revised figure 3 to indicate the significant posthocs.

**…together with a graph illustrating this secondary analysis.**

Figure 4 has been added to illustrate the posthoc test

***Line 175 : As I understand, the ranges mentioned here are inter-individual range of the number of trials in each temporal bin, may be it should be mentioned.**

Line 180 edited as suggested

***Line 215 : « The finding that that », please correct the typo**

Corrected

***Line 215 : « many have implications », please correct the typo, many should be « may »**

Corrected

***Line 216 : spell out what AMD initials mean, age macular degeneration ? This is a deficit concerning central vision, so I do not clearly see how the result of this paper could be related to it ? Speculation about hemianopia is more relevant and would be interesting to test. Furthermore, hemianopia could come from different lesions, at the level of V1 or at the level of the optic nerve after the optic chiasma. It could be interesting to contrast these two levels.**

"The original idea of including discussion of AMD (age macular degeneration) was to ask what happens to presaccadic attention when the fovea is blind. On reflection we agree this is probably not that relevant to the current experiment, as the issue with AMD is retinal not cortical. We have therefore removed reference to AMD.

The suggestion to compare hemianopes with V1 vs damage to the optic chiasm is really interesting. It seems to us that the patients with post-chiasmatic optic nerve damage have a deficit that is most similar to the blindspot, as there remains a retinotopically organised region in V1 that does not receive any visual input. It is however less certain what the predictions are for patients with cortical damage. In this case the cortical retinotopy will have broken down, and it is not clear how this will affect the remapping process. We have added a couple of lines to the discussion to make this point.

**Reviewer #2:**  **It's a clever experiment, well-designed and presented, and the results seem clear enough. I have a few suggestions for revision, mostly cosmetic:**

Thank you for the positive feedback

**1) It would generally help to have a bit more information in the figure captions, though I was mostly able to figure out what was shown. The exception was figure 1. What is shown in the left panel? How large a portion of the visual field is represented by each square in the right panel? The text doesn’t refer to the figure at all.**

The left and right panel show the same data, but the right panel illustrates performance at each of the tested locations, whereas the left panel shows a probability plot. As the information in the left panel is redundant this panel has been removed. Details on the size of the square have been added to the caption, and a reference to the figure added to line 119

**2) What was the contrast of the discrimination target? How was it chosen?**

The target was white on a black background with approximately 100% contrast. We selected a high contrast target as we needed something that would be discriminable even at quite large distances from fovea, but would also fall fully within the blindspot. The best way to achieve this was to use relatively small but high contrast target.

**3) My main suggestion is to include more information about how the timing of events in the experiment was verified. This is obviously critical, because the reported results can arise trivially if the display of the probe persists into the postsaccadic period. This can happen in two ways:
a. Filtering/smoothing of the eye position traces often lead to inaccurate estimates of saccade latency. Please indicate how this was done.**

We agree timing is critical. The selection algorithm smoothed over 4 samples and used a criteria of a minimum velocity of 70 d/s and duration of 20ms to detect eye-movements.

**b. Persistence of the stimulus is a well-known problem, particular with bright stimuli on a dark background in CRT displays (see the back and forth on this topic in the early 1980s, e.g., Irwin, Yantis, and Jonides (1983)). I doubt this is a problem for the current work, because the authors wisely used a mask to eliminate any potential persistence. But it seems like there were some trials in which the offset of the probe occurred very close to the saccade, and it would be good to make sure that small timing errors did not influence the results.**

We excluded any trials where the saccade began before the onset of the mask. Given that a 5 degree saccade has a duration of 20-30ms, we feel this is rather conservative selection criterion that guarantees that the blindspot cannot physically have overlapped the target position on any trial. Nevertheless, we also looked at the 123 trials in which the saccade began between 1 and 10ms after the onset of the mask, of which 23 were trials where the target appeared in the Blindspot FF. Mean accuracy in the blindspot FF was 60% on these trials. Given the very small number of trials we feel confident that, even if the occasional trial with small timing errors slipped through the filtering, the main finding cannot be explained away by these trials. It is also worth noting that our second analysis indicates that perceptual impairment is present in the distal bin, which cannot possibly be affected by small errors in timing that would mean the blindspot was occluding the target position before the mask appeared.

**4) Related to the previous point, I didn’t entirely understand the timing. What was the actual distribution of probe timing relative to saccade onset? The bins described in the paragraph around line 171 seem arbitrary and not entirely consistent with the reported range of saccade latencies and the experiment timing shown in Figure 2.**

We thank the reviewer for pointing this out. We had omitted a line explaining the inclusion criteria for trials in the first analysis, which we agree makes the discussion of the timing a bit hard to understand. To clarify: For the first analysis we excluded all trials where the saccade began before the mask was presented. We included trials where a saccade was executed between 0 and 300 ms of the onset of the mask. Note this is slightly different to the second analysis, in which we included trials in which saccades were executed up to 340ms after saccade onset. The reviewer is correct that the bins were somewhat arbitrary as we were aiming for bins of the same temporal size and a minimum of 20 trials per bin. We have revised the results section (lines 165-187) to try and make this more explicit to the reader.

**5) The authors might be interested in a few older papers on visual receptive fields covering the blind spot from Komatsu’s group – see for example Matsumoto and Komatsu (2005).**

We thank the reviewer for this suggestion and have added a line about this study to the discussion
